# Characterization of capacitive electromyography biomedical sensor insulated with porous medical bandages

**DOI:** 10.1038/s41598-020-71709-0

**Published:** 2020-09-10

**Authors:** Charn Loong Ng, Mamun Bin Ibne Reaz, Maria Liz Crespo, Andres Cicuttin, Muhammad Enamul Hoque Chowdhury

**Affiliations:** 1 Department of Electrical, Electronic and Systems Engineering, Faculty of Engineering and Built Environment, Universiti Kebangsaan Malaysia, 43600 Bangi, Selangor Darul Ehsan Malaysia; 2 Multidisciplinary Laboratory, International Centre for Theoretical Physics (ICTP), Via Beirut, 31-34151 Trieste, Italy; 3 Department of Electrical Engineering, Qatar University, 2713 Doha, Qatar

**Keywords:** Biomedical engineering, Sensors and biosensors, Electromyography - EMG

## Abstract

A capacitive electromyography (cEMG) biomedical sensor measures the EMG signal from human body through capacitive coupling methodology. It has the flexibility to be insulated by different types of materials. Each type of insulator will yield a unique skin–electrode capacitance which determine the performance of a cEMG biomedical sensor. Most of the insulator being explored are solid and non-breathable which cause perspiration in a long-term EMG measurement process. This research aims to explore the porous medical bandages such as micropore, gauze, and crepe bandage to be used as an insulator of a cEMG biomedical sensor. These materials are breathable and hypoallergenic. Their unique properties and characteristics have been reviewed respectively. A 50 Hz digital notch filter was developed and implemented in the EMG measurement system design to further enhance the performance of these porous medical bandage insulated cEMG biomedical sensors. A series of experimental verifications such as noise floor characterization, EMG signals measurement, and performance correlation were done on all these sensors. The micropore insulated cEMG biomedical sensor yielded the lowest noise floor amplitude of 2.44 mV and achieved the highest correlation coefficient result in comparison with the EMG signals captured by the conventional wet contact electrode.

## Introduction

The electromyography (EMG) signal is a quasi-periodic bioelectrical signal that generated during a muscle contraction. This signal contains useful information for a wide range of applications such as clinical diagnosis of musculoskeletal diseases (MSD), healthcare and rehabilitation, and prothesis controller. This alternating bioelectrical signal appear in a burst form which can be measured with the capacitive coupling methodology. The advantages of a capacitive electromyography (cEMG) biomedical sensor are simple donning and doffing process, not required conductive gel, and suitable for long-term monitoring application in comparison with the conventional wet contact electrode (Ag–AgCl)^1^. Different types of insulator such as metal oxide, solder mask, adhesive tape, and textile have been explored by researchers to improve the design and flexibility of the cEMG biomedical sensor^2,3^. However, most of the insulators are solid and non-breathable material which will cause perspiration after a long-term electromyography (EMG) measurement. This phenomenal will change the skin–electrode capacitance value and eventually alter the performance of a cEMG biomedical sensor^4^.

Porous medical bandages are commonly used in the medical treatment process such as wound treatment. They are hypoallergenic, clinical approved material, and commonly available in the market which made them a good option to be used as an insulator of a cEMG biomedical sensor. Having these medical grade bandages as a skin–electrode interface also can help to accelerate the adoption of a capacitive measurement technology in the clinical and healthcare sector. The primary focus of this journal is to analyze the characteristic and performance of a cEMG biomedical sensor that insulated by different types of porous medical bandage. This research paper explored three types of porous medical bandage which are micropore, gauze, and crepe bandage. Each porous medical bandage has a unique porosity, thickness, and base material which will yield a unique skin–electrode capacitance when using them to insulate the cEMG biomedical sensor. A literature review, theoretical analysis, and experimental characterization were performed to understand the properties of these porous medical bandages. The cEMG biomedical sensor was insulated by these porous medical bandage samples to measure the EMG signals from the bicep brachii muscle of two subjects. The performance of these sensors were benchmarked with the conventional wet contact electrode (Ag–AgCl). A 50 Hz digital notch filter is added in the post signal processing process to improve the signal-to-noise ratio of the porous medical bandages insulated cEMG biomedical sensor^5,6^.

## Method and materials

### Porous medical bandage properties and characteristics

There are a variety of porous medical bandages being used in the medical treatment sector. Micropore, gauze, and crepe bandage were selected in this research because each of them has a unique standard of porosity. Table 1 summarizes the properties of all three porous medical bandages.

**Table 1:** Properties of the porous medical bandages.

Porous material	Micropore	Gauze	Crepe Bandage
Base material	Paper	Cotton	Cotton
Porous dimension	Micro-meter	19 × 15 mesh	Warp not less than 170, Weft not less than 78
Sterility	Non-sterile	Non-sterile	Non-sterile
Physical characteristic	Breathable, hypoallergenic, adhesive	Breathable, hypoallergenic	Breathable, hypoallergenic, stretchable
Manufacturer	3M	Nur Care, Gauze Roll, NC1750	Nur Care, elastic creape bandage, NC1730
Sample image of the porous materials			

Micropore is an adhesive tape that made of paper. It has a porous dimension at the range of micro-meter which creates a good electrical isolation between the cEMG biomedical sensor’s electrode and human body with minimal air gap^7^. Gauze is a non-adhesive medical bandage that made of cotton or a mixture of cotton^8^. The sample use in this research has a standard 19 × 15 mesh porous dimension which has an air gap between the thread that is much larger than the micropore. Crepe bandage is a stretchable bandage that made of cotton as well. It has the porous dimension define at warp not less than 170 and weft not less than 78^9^. Table 1 shows the image of the porous medical bandage samples where the air gap of gauze and crepe bandage are clearly visible while the micropore is invisible. All of these porous medical bandages are hypoallergenic and breathable which is suitable for different skin condition and long-term EMG signal monitoring application. These porous medical bandage samples under test are non-sterile.

### cEMG biomedical sensor specification

With different base materials, thickness, and porosity, these medical bandages are expected to have a unique relative permittivity value. Due to the porosity characteristic and large air gap, these medical bandages are having a relative permittivity close to 1. The relative permittivity of each medical bandage sample was measured using parallel-plate capacitive model^10–12^. The skin–electrode capacitance of the cEMG biomedical sensor insulated by different porous medical bandages were calculated using Eq. (1) and recorded in Table 2.

**Table 2:** Skin–electrode capacitance of the cEMG biomedical insulated by porous materials.

Porous insulators	Electrode area size, $A$ (${\mathrm{mm}}^{2}$)	Relative permittivity, ${\varepsilon }_{r}$	Thickness, $d$ (mm)	Skin–electrode capacitance, ${C}_{s}$ (pF)^a^
Micropore	510	1.920	0.16	54.173
Gauze	510	2.041	0.45	20.476
Crepe Bandage	510	2.753	0.88	14.123

^a^Calculated using Eq. (1) with the ${ \varepsilon }_{0}=8.854 \times {10}^{-12} \mathrm{F}/\mathrm{m}$.

1 $${C}_{s} = {\frac{{\varepsilon }_{0}{\varepsilon }_{r} A}{d}}$$

where ${\varepsilon }_{0}$ is the vacuum permittivity, $8.854 \times {10}^{-12} F/m$, ${\varepsilon }_{r}$ is the relative permittivity, $A$ is the area size of the electrode, $d$ is the thickness of the porous insulator.

**Figure 1:**
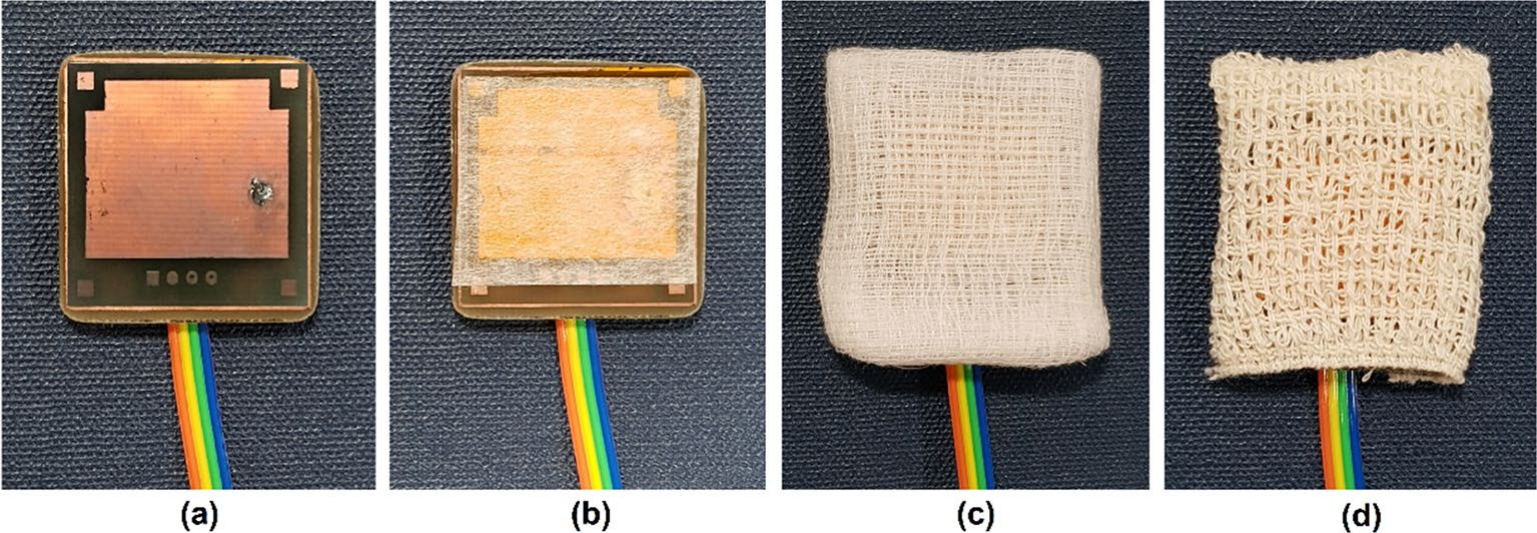
The cEMG biomedical sensor insulated with porous materials. **(a)** Original cEMG biomedical sensor without insulator. cEMG biomedical sensor insulated by **(a)** micropore, **(b)** gauze, and **(c)** crepe bandage.

Micropore has the highest skin–electrode capacitance which is 54.173 pF while the crepe bandage has the lowest skin–electrode capacitance at 14.123 pF. Gauze has a skin–electrode capacitance value between the micropore and crepe bandage which is 20.476 pF. Since the skin–electrode capacitance value is inversely proportional to the noise floor of a cEMG biomedical sensor, crepe bandage is expected to have the highest noise floor follow by gauze and micropore^4^. Figure 1 shows the cEMG biomedical sensor insulated by micropore, gauze, and crepe bandage.

### Capacitive electromyography measurement system

**Figure 2:**
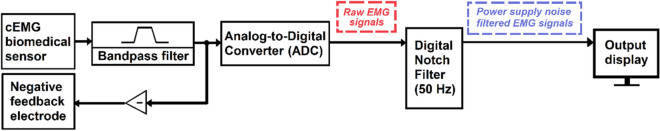
Architecture of the cEMG measurement system.

The porous medical bandage insulated cEMG biomedical sensor is integrated with a measurement system to evaluate the performance. The architecture of the measurement system is illustrated in Fig. 2. The detail electrical design of the cEMG biomedical sensor, bandpass filter, and negative feedback circuit were presented in^13^. The cEMG biomedical sensor contains a frontend buffer to drive the EMG signals across a 1 m cable. Both the cEMG biomedical sensor and the bandpass filter have a unity gain to capture the raw EMG signals. The bandpass filter has a passband frequency between 10 and 300 Hz because it is the dominant frequency range of an EMG signal^8^. An NI myDAQ is used as an analog-to-digital converter (ADC) to digitize the EMG signals. In order to improve the signal-to-noise ratio of the measurement result, the raw EMG signals data are post-processed by a 50 Hz digital notch filter to attenuate the power line noise that couple into the measurement result.

### 50 Hz digital notch filter design parameter

**Figure 3:**
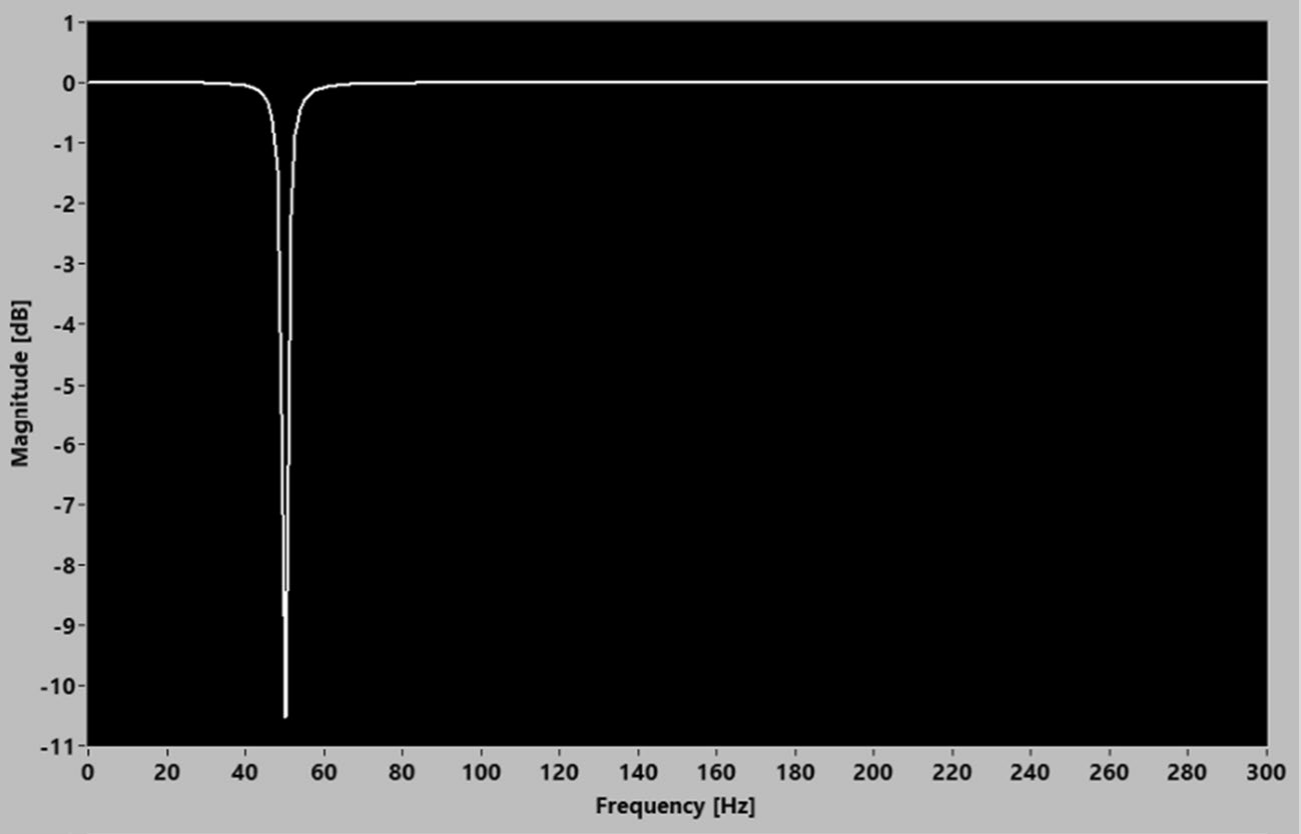
Magnitude response of the 50 Hz digital notch filter.

A digital notch filter is implemented to attenuate the targeted 50 Hz power line noise^14,15^. The advantage of a digital filter is the flexibility to change its filtering characteristic and performance without incurring significant development time and cost. This research utilized the NI LabVIEW infinite impulse response (IIR) second order notch filter VI to design a 50 Hz digital notch filter to suppress the 50 Hz power supply noise in the measurement results. The transfer function of the IIR second order digital notch filter is described in Eq. (2) while the design parameters of the 50 Hz digital notch filter are listed in Table 3. Figure 3 shows the magnitude response of the implemented 50 Hz notch filter.

**Table 3:** Design parameter of the 50 Hz IIR digital notch filter VI.

Input parameter	Values
Filter type	Notch
Center frequency, f0 (Hz)	50
Q factor	20
Sampling frequency, fs (Hz)	600

2 $$H\left(z\right)= \frac{{b}_{0}\left(1+ \frac{{b}_{1}}{{b}_{0}}{z}^{-1}+{z}^{-2}\right)}{1+ {a}_{0}{z}^{-1} + {a}_{1}{z}^{-2}}$$

where ${a}_{0} , {a}_{1}, {b}_{0}, {b}_{1}$ are the filter coefficient.

### Experimental setup

The characteristic and performance of a cEMG biomedical sensor insulated by micropore, gauze, and crepe bandage were evaluated in different experiments. These cEMG biomedical sensors were tested on two healthy subjects aged between 20 and 40 years old to analyse the consistency of the performance. This study was approved by the Research Ethics Committee (REC) of Universiti Kebangsaan Malaysia. All methods and experiments were performed in accordance with relevant guidelines and regulations. Informed consent was obtained from each subject prior to their participation in the experiment.

A wet contact electrode (Ag–AgCl) is the gold standard of a surface EMG measurement^16^. It was used to benchmark the performance of these porous medical bandage insulated cEMG biomedical sensors^17^. The cEMG biomedical sensor and wet contact electrode were placed in close proximity on the bicep brachii of a subject to capture the same EMG signal source generated during a dynamic muscle contraction. The bicep brachii is an ideal muscle unit because it is a large single muscle that can be control by the subject consistently.

## Results and discussion

### Noise floor characterization

**Figure 4:**
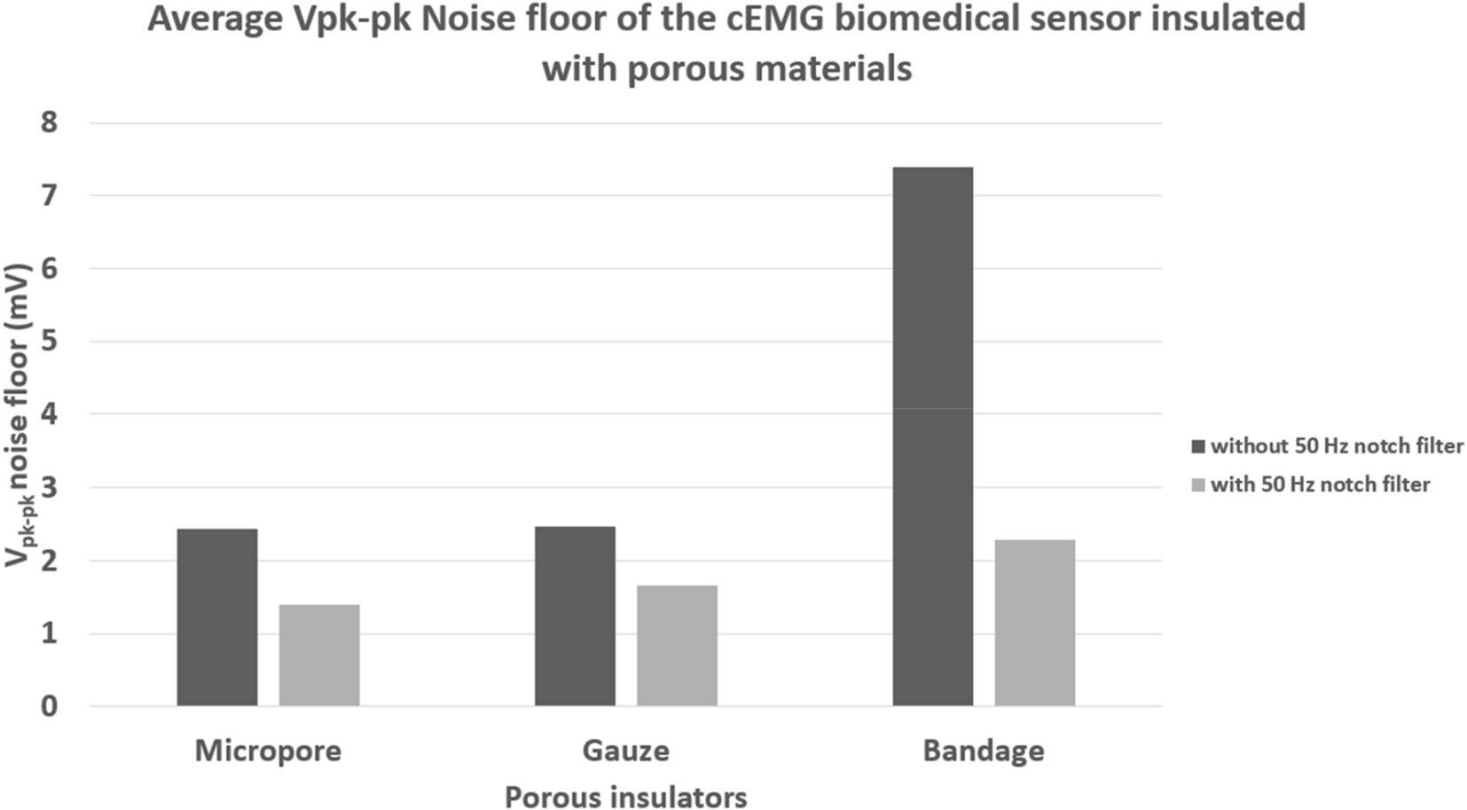
The average noise floor amplitude recorded from the cEMG biomedical sensor insulated with different porous materials.

The noise floor characterization test measured the baseline result of the porous medical bandages insulated cEMG biomedical sensor. Since the EMG signal is a weak bioelectrical signal range from 0.05 to 5 mV, the noise floor of these sensors ought to be as low as possible to maximize the signal-to-noise ratio^2^. In this test, the subjects’ bicep brachii were required to stay idle and at ease during the measurement, to ensure no EMG signals were generated. Figure 4 shows the average peak-to-peak noise floor amplitude $({V}_{pk-pk})$ measured from the two subjects. The micropore and the gauze recorded a similar noise amplitude of 2.44 mV and 2.47 mV. Crepe bandage recorded the highest noise floor of 7.386 mV.

**Figure 5:**
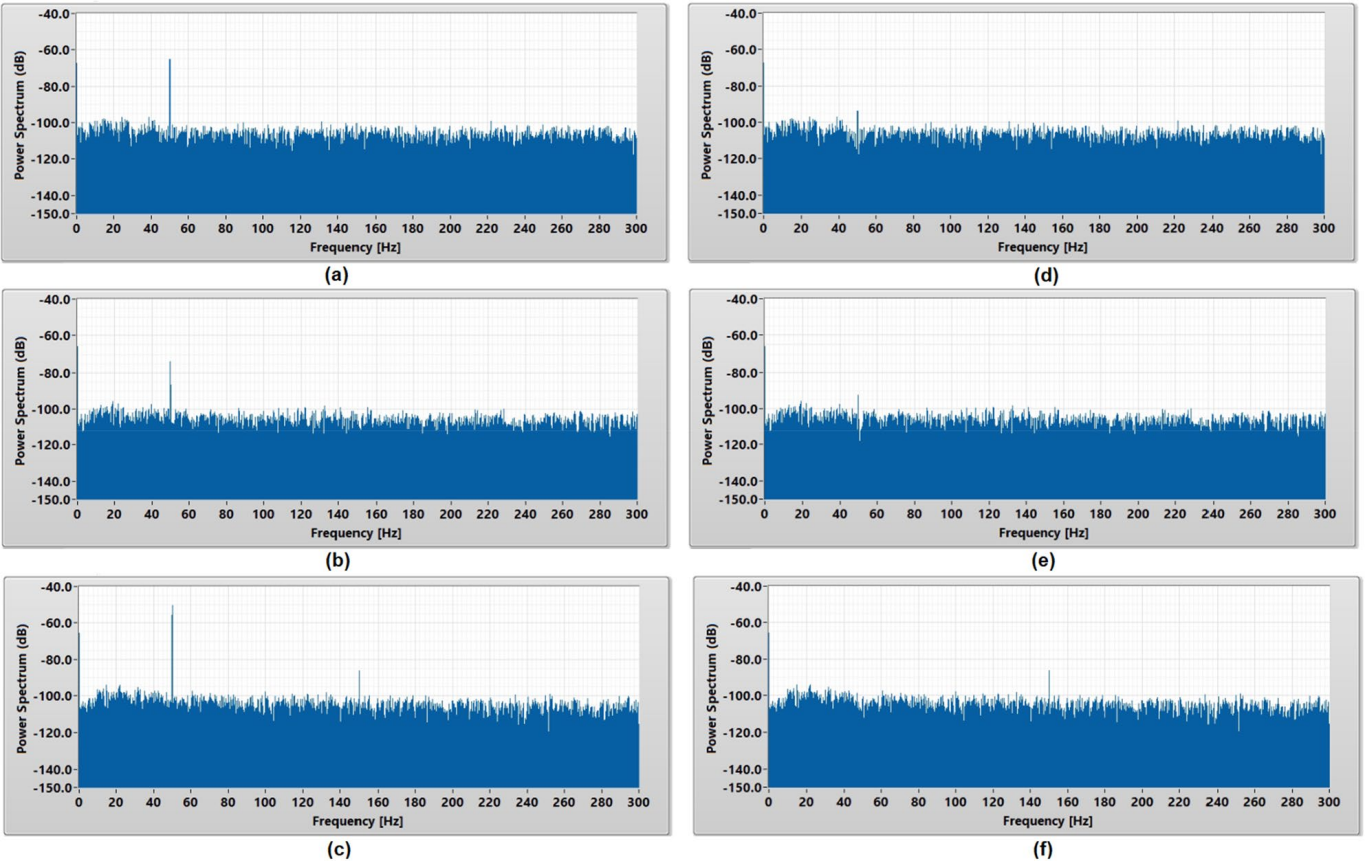
Power spectrum analysis of the noise floor captured using **(a)** micropore, **(b)** gauze, and **(c)** crepe bandage without 50 Hz digital notch filter and **(d)** micropore, **(e)** gauze, and **(f)** crepe bandage with 50 Hz digital notch filter.

The power spectrum analysis of the noise floor in Fig. 5 shows that the 50 Hz power line noise was the main contributor of the noise floor for all types of cEMG biomedical sensors. Micropore insulated cEMG biomedical sensor recorded − 65 dB, gauze insulated cEMG biomedical sensor recorded − 73 dB, and crepe bandage insulated cEMG biomedical sensor recorded − 50 dB. The crepe bandage also observed a harmonic frequency spike of − 86 dB at 150 Hz while other porous materials do not observe a harmonic frequency spike.

Since the experiment was done in an environment without electromagnetic wave shielding, the surrounding noises such as 50 Hz power line noise was expected to couple into the measurement results. Generally, a cEMG biomedical sensor has a high input impedance characteristic, thus it is highly susceptible to the environmental noises. The porous material that formed a lower skin–electrode capacitance will have a higher input impedance and eventually becomes the dominant factor of a cEMG biomedical sensor recording a higher noise floor. The 1-m cable connecting the cEMG biomedical sensor and the bandpass filter module is a single core ribbon cable insulated with PVC material. Since this cable is a non-shielded cable, thus it is also susceptible to the electromagnetic noise in the surrounding. This ribbon cable contains four parallel electrical lines which arrange in the sequence of + 15 V, − 15 V, analog ground, and the measured EMG signal. The EMG signal line is placed next to the analog ground and the experimental setup is powered by battery to reduce the interference of the 50 Hz power line noise to the EMG measurement results. Generally, a cable that is longer than λ/20 is consider electrically long and started to act as a reception antenna^18^. The Eq. (3) can be used to determine the wavelength of the highest frequency of concern.

3 $$\lambda = \frac{c}{f}$$

where λ is the wavelength in meter. *ƒ* is the target frequency in Hz. c is the speed of light which is $3 \times {10}^{8}  {\rm {m/s}}$.

For a 50 Hz noise signal, the value of λ/20 is 300 km. Therefore, a 50 Hz power line noise can hardly induce into the 1-m cable and corrupt the EMG signals. However, any high frequency noises which is more than 15 MHz is still possible to induce into the 1-m cable. The bandpass filter which has a passband frequency between 10 and 300 Hz will attenuate these high frequency noises in the EMG measurement results.

A 50 Hz digital notch filter was designed and implemented to attenuate the 50 Hz power line noise in the measurement to improve the overall noise floor of a porous medical bandage insulated cEMG biomedical sensor. The power spectrum analysis in Fig. 5 shows the significant reduction of 50 Hz noise in the baseline measurement after implemented the digital notch filter. Figure 4 shows that the 50 Hz digital notch filter has successfully reduced the noise floor of the micropore insulated cEMG biomedical sensor from 2.44 to 1.39 mV and gauze insulated cEMG biomedical sensor from 2.47 to 1.65 mV. The bandage insulated cEMG biomedical sensor has the greatest noise floor amplitude reduction from 7.39 to 2.28 mV. With the 50 Hz digital filter implemented, the difference of the noise floor amplitude among the porous materials has reduced from 4.94 to 0.89 mV, which makes them have a relatively similar baseline result.

### EMG burst signals measurement

**Figure 6:**
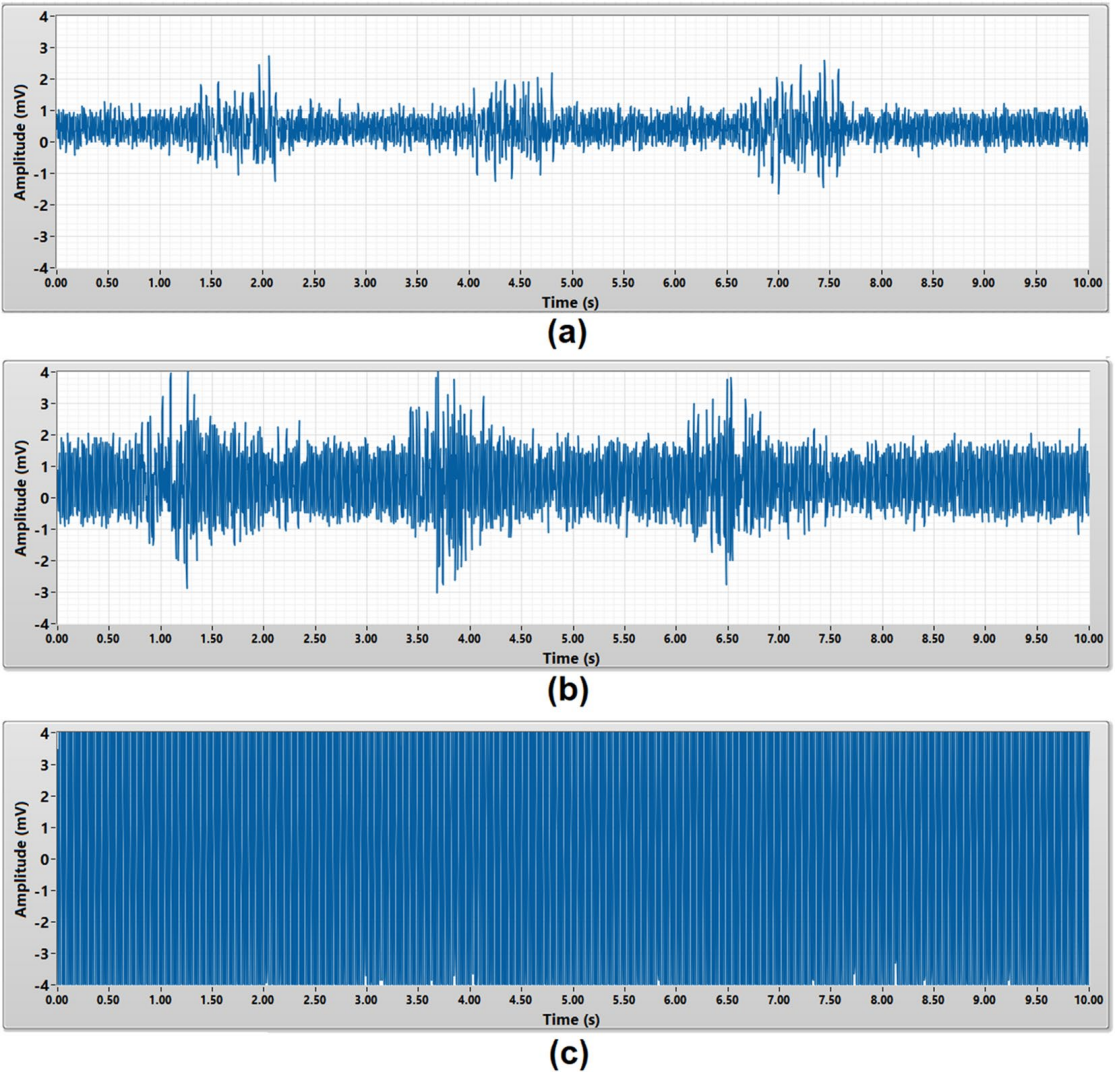
Raw EMG burst signals acquired by **(a)** micropore, **(b)** gauze, **(c)** crepe bandage without 50 Hz digital notch filter.

**Figure 7:**
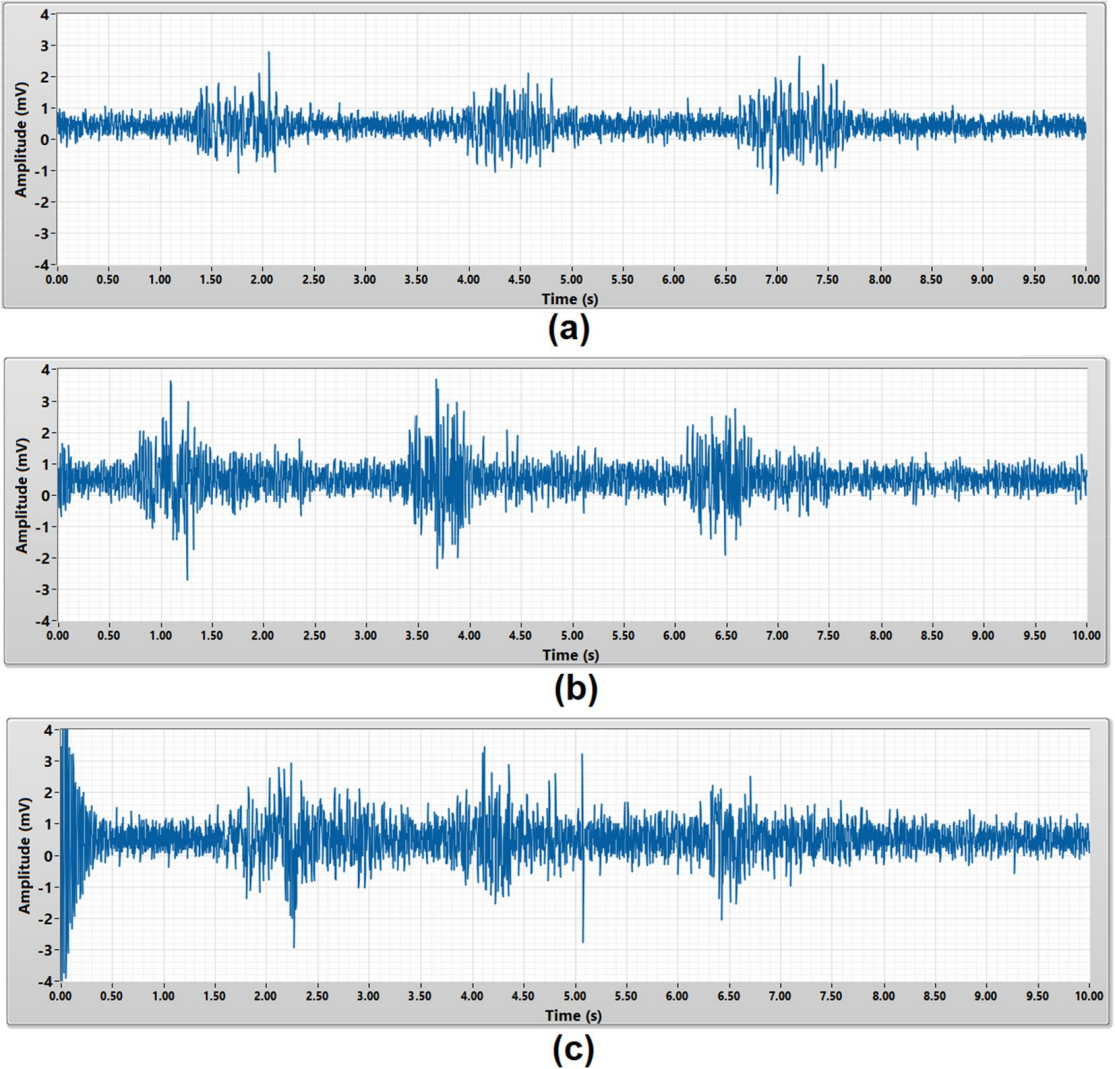
EMG burst signals acquired by **(a)** micropore, **(b)** gauze, **(c)** crepe bandage with 50 Hz digital notch filter.

The EMG burst signals measurement was to validate the capability of the porous medical bandage insulated cEMG biomedical sensor to measure a complete EMG burst signals from a muscle. Within a 10 s duration, each subject was required to perform dynamic contract of their bicep brachii for three times to generate the EMG burst signals. The amplitude of an EMG burst signals is expected to be around 0.05 mV to 5 mV^2^. All three types of the porous material were tested on each subject. Figures 6 and 7 present the raw and 50 Hz noise filtered EMG burst signals captured from subject A using different porous medical bandage as an insulator. Each EMG burst signal is around 1 s duration.

Since the micropore insulated cEMG biomedical sensor has the lowest noise floor, the three EMG burst signals were clearly observed in Fig. 6a. The gauze insulated cEMG biomedical sensor has higher noise floor compare to micropore insulated cEMG biomedical sensor, thus the EMG burst signals is not clearly seen in Fig. 6b. As for crepe bandage insulated cEMG biomedical sensor, the noise floor amplitude is much higher than the EMG signals, thus the frontend buffer is saturated and unable to observe the EMG burst signals in Fig. 6c. Without required post signal processing, micropore insulated cEMG biomedical sensor yield the best measurement result with the highest signal-to-noise ratio.

In order to improve the overall porous medical bandage insulated cEMG biomedical sensor’s performance, a 50 Hz digital notch filter was implemented in the post signal processing stage to reduce the power line noise in the measurement results. The 50 Hz digital notch filter has a 0.5 s settling transient during the start-up stage. The micropore insulated cEMG biomedical sensor with 50 Hz notch filter yielded a better signal-to-noise ratio measurement result as shown in Fig. 7a. All three EMG burst signals were clearly observed. The EMG burst signals measured by gauze insulated cEMG biomedical sensor also can be seen clearly after implementing the 50 Hz digital notch filter. The 50 Hz power line noise is greatly reduced in the crepe bandage insulated cEMG biomedical sensor measurement results after the implementing the 50 Hz digital notch filter. Three EMG burst signals can be observed in Fig. 7c.

### Performance evaluation

The performance evaluation validated the consistency, accuracy, and repeatability of the porous medical bandage insulated cEMG biomedical sensors in EMG signal measurement. The performance of these sensors was benchmarked with the conventional wet contact electrode (Ag–AgCl) which is a gold standard in clinical applications. In this experiment, all two subjects were required to contract their bicep brachii for 1 s to generate the EMG signal. The same EMG signal source were measured by both sensors. This test was repeated five times on every subject, to guarantee the repeatability and reliability of the measurement result.

**Figure 8:**
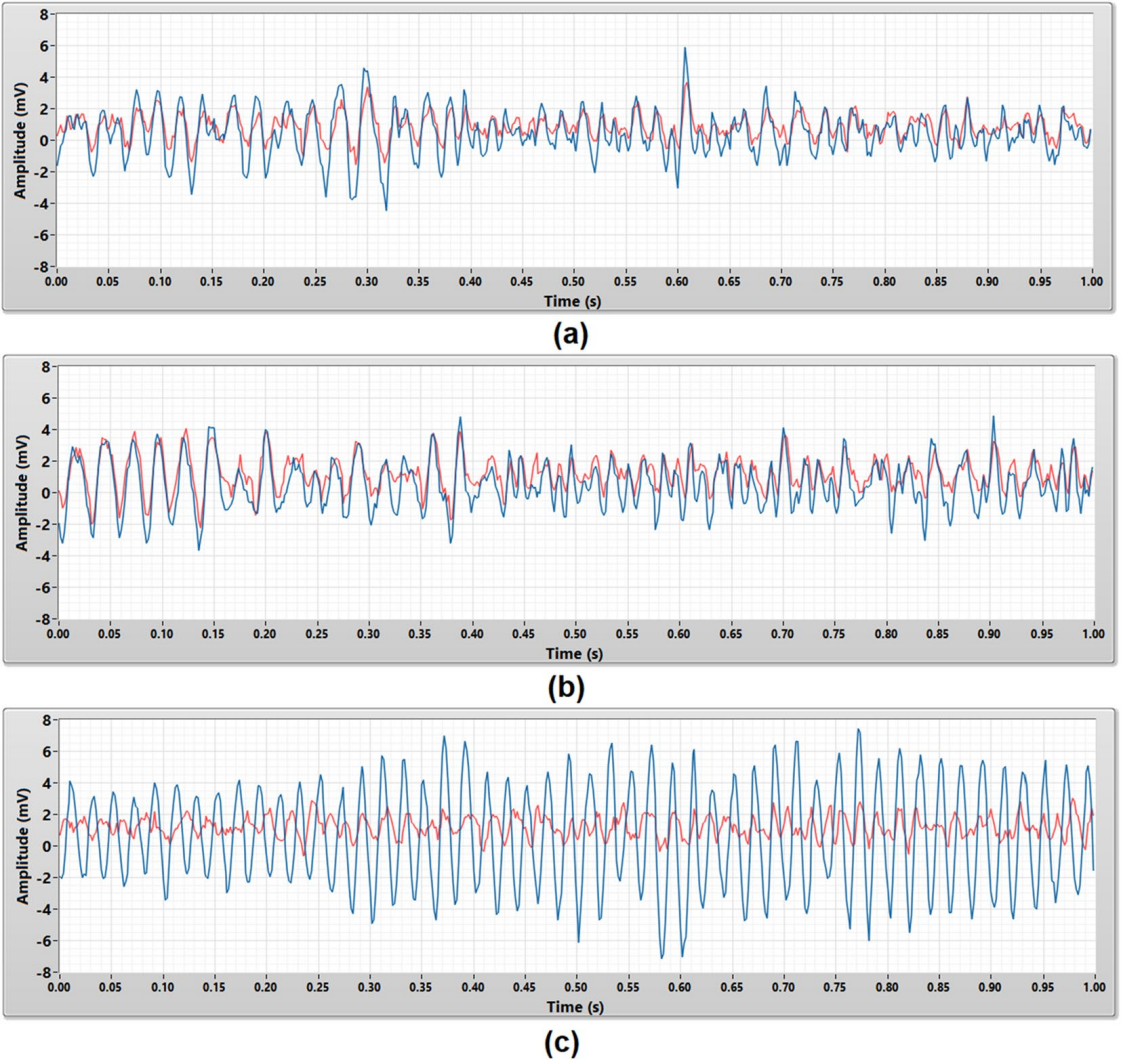
The raw EMG signals that acquired using the wet contact electrode (red) and the porous medical bandage insulated cEMG biomedical sensor (blue) in 1 s duration. The porous medical bandages are **(a)** micropore, **(b)** gauze, and **(c)** crepe bandage.

Figure 8 shows the 1 s raw EMG signals captured by the micropore, gauze, and crepe bandage insulated cEMG biomedical sensor. EMG signals captured by the micropore and gauze insulated cEMG biomedical sensor have a similar signal pattern and amplitude compare to the EMG signals captured by the wet contact electrode. The crepe bandage insulated cEMG biomedical sensor was corrupted by the power line noise, therefore the signal captured is a 50 Hz periodical sine wave with uneven amplitude and it is completely different from the EMG signal measured by the wet contact electrode.

**Figure 9:**
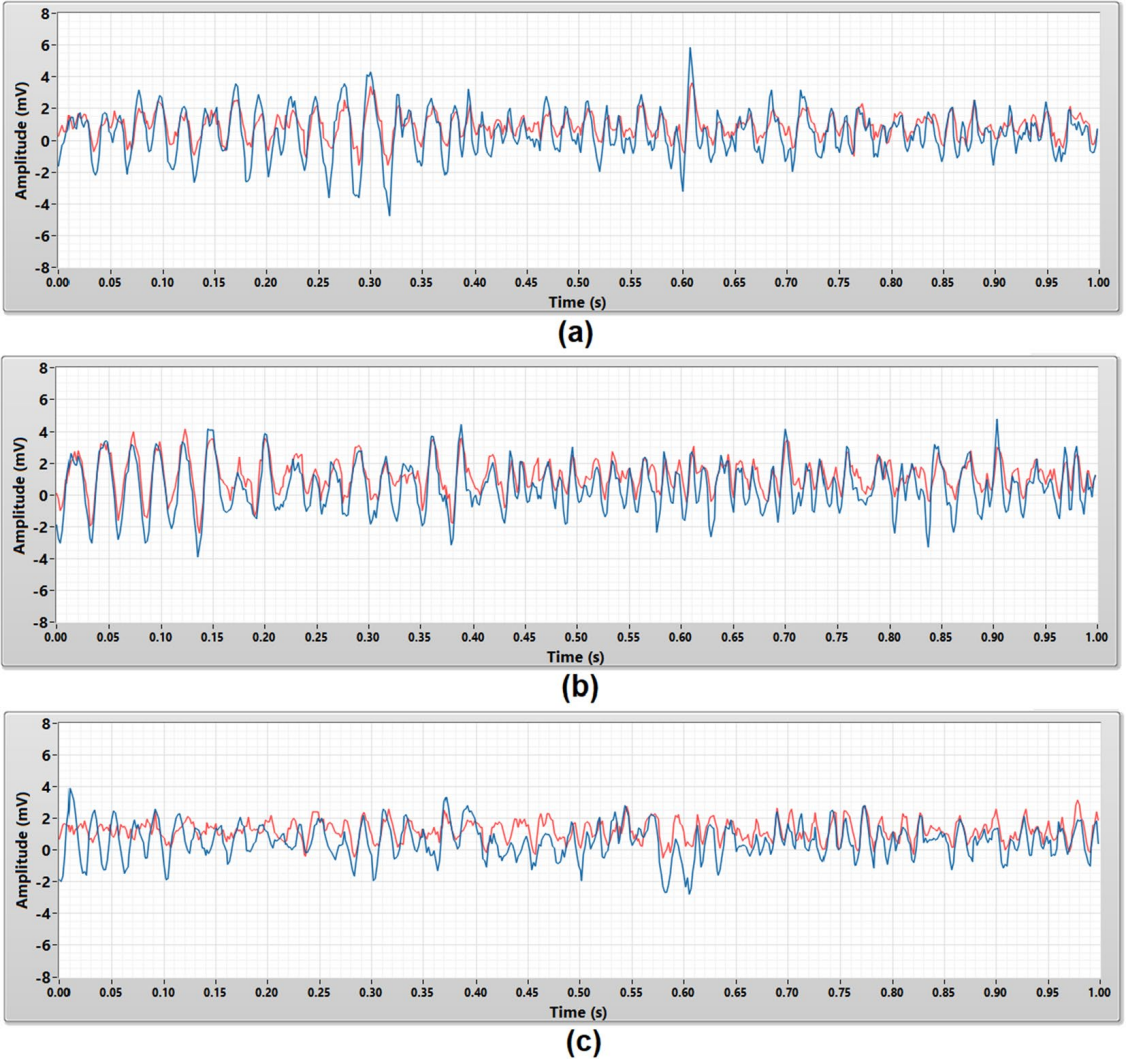
The 50 Hz noise filtered EMG signals that acquired using the wet contact electrode (red) and the porous medical bandage insulated cEMG biomedical sensor (blue) in 1 s duration. The porous medical bandages are **(a)** micropore, **(b)** gauze, and **(c)** crepe bandage.

These raw EMG signals were post-processed by a 50 Hz digital notch filter to further evaluate the measurement results. Figure 9 shows the raw EMG signals in Fig. 8 filtered by the 50 Hz digital notch filter. Both micropore and gauze insulated cEMG biomedical sensor’s measurement results had a minimal improvement. Their EMG measurement results were still closely match with the EMG signals measured by the wet contact electrode. The EMG signals measured by the crepe bandage insulated cEMG biomedical sensor is no longer dominated by the power line noise (50 Hz periodical sine wave) after implemented the 50 Hz digital notch filter. Its pattern and amplitude were similar to the EMG measurement result captured by the wet contact electrode but not a close match.

**Figure 10:**
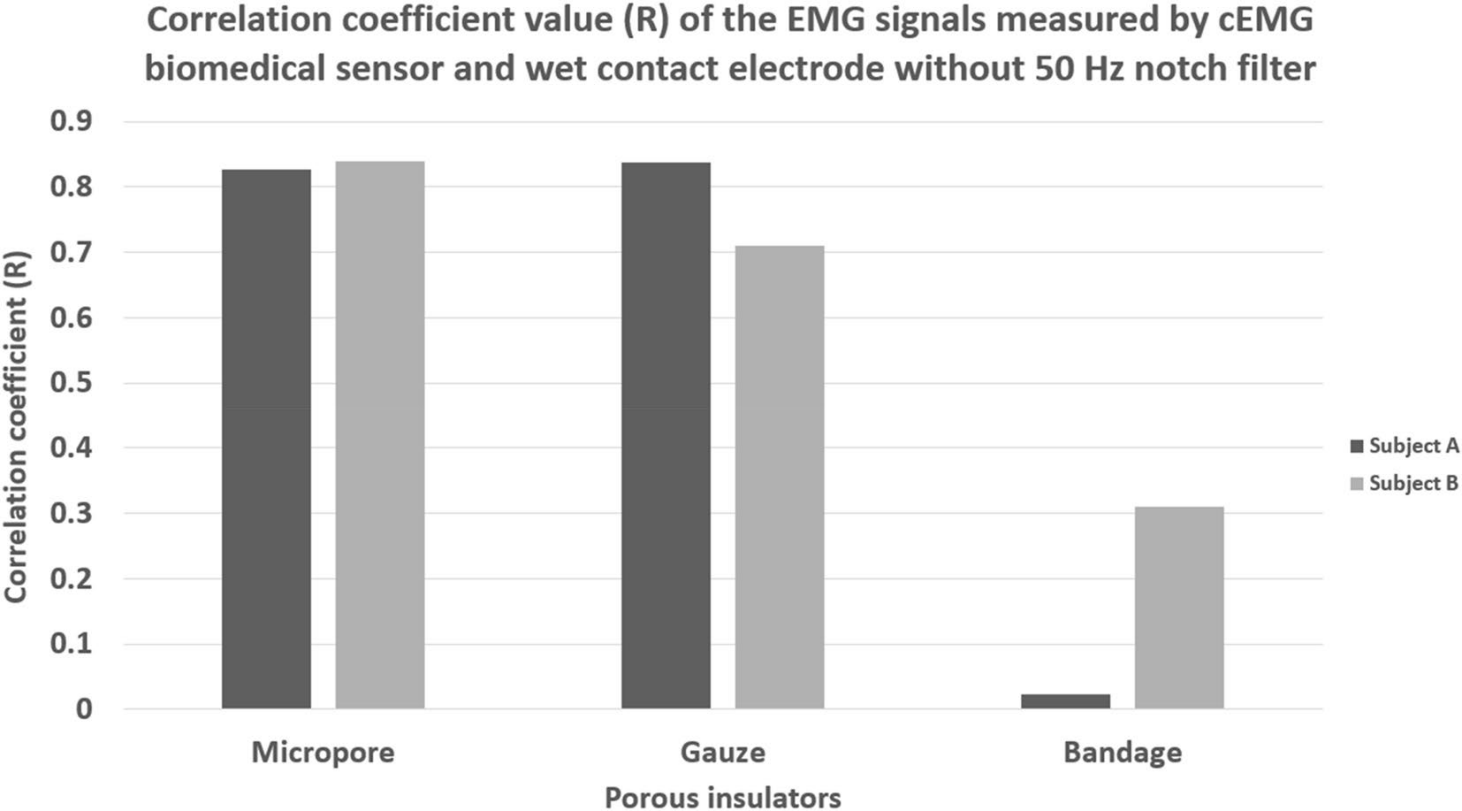
Correlation coefficient value of the raw EMG signal measure using wet contact electrode and porous medical bandage insulated cEMG biomedical sensor without 50 Hz digital notch filter.

The correlation coefficient (R) values of the EMG signals measured by the porous medical bandage insulated cEMG biomedical sensor and wet contact electrode were calculated. The average correlation coefficient values of the raw EMG signals of each subject were shown in Fig. 10. The correlation coefficient values were not expected to achieve 1.0 because both cEMG biomedical sensor and wet contact electrode were not possible to place at the exact same location. The micropore insulated cEMG biomedical sensor yielded a consistent measurement results between the subject A and subject B. High average correlation coefficient value of 0.83 and 0.84 were shown between the EMG signals measured by the wet contact electrode and the micropore insulated cEMG biomedical sensor. The EMG signals measured by the gauze insulated cEMG biomedical sensor have 0.84 and 0.71 correlation coefficient value with the wet contact electrode. The gauze insulated cEMG biomedical sensor yielded a relatively high correlation coefficient values, but the results were not consistent between the two subjects. The EMG signals measured by the crepe bandage insulated cEMG biomedical sensor and the wet contact electrode were uncorrelated which yielded the correlation coefficient values of 0.02 and 0.31 on subject A and subject B. Since the crepe bandage insulated cEMG biomedical sensor was corrupted by the power line noise as shown in Figs. 6 and 8, an uncorrelated result was expected.

**Figure 11:**
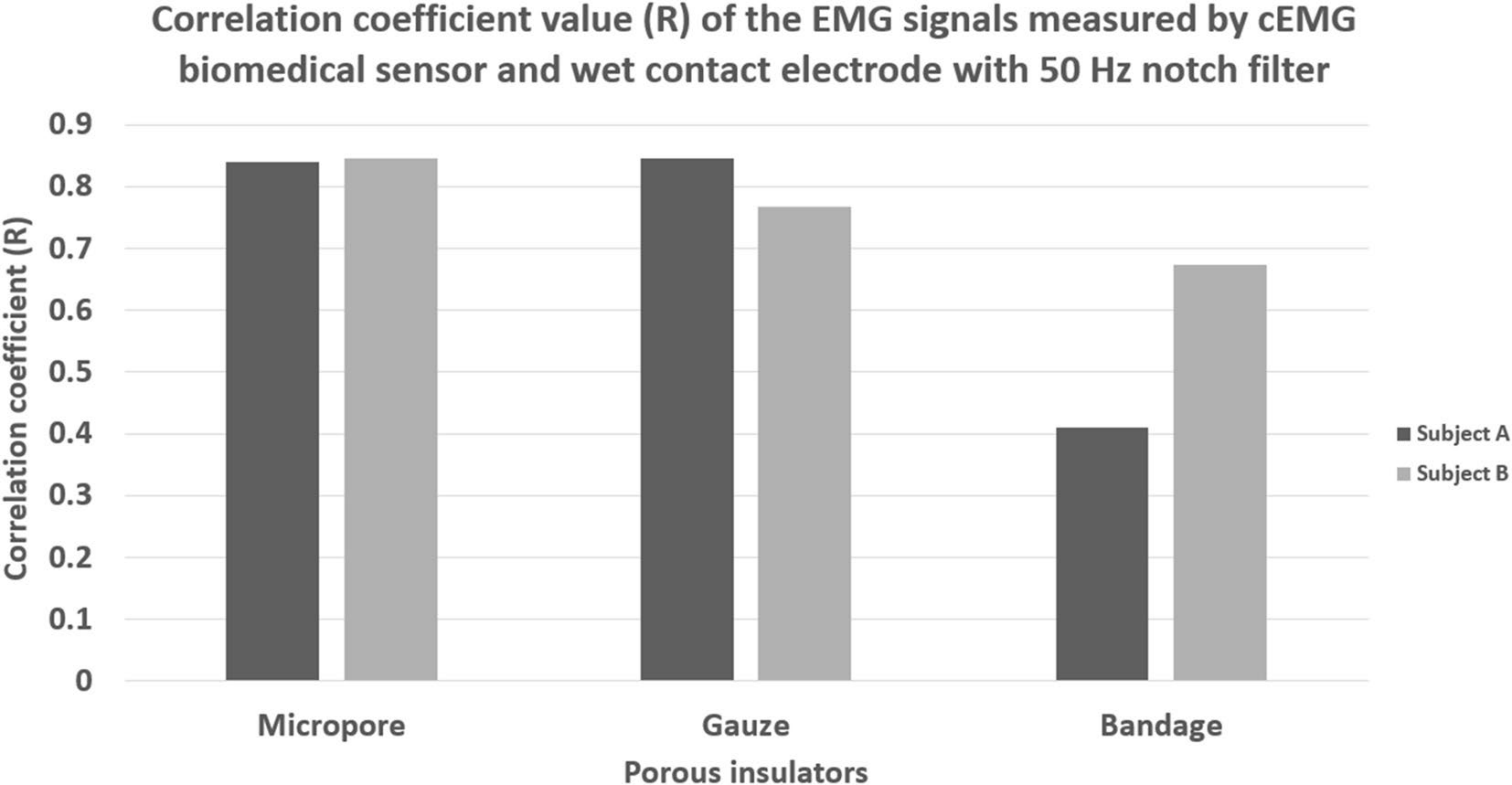
Correlation coefficient value of the EMG signal measure using wet contact electrode and porous medical bandage insulated cEMG biomedical sensor with 50 Hz digital notch filter.

The correlation coefficient value of the EMG signals post-processed by the 50 Hz digital notch filter were calculated as well to evaluate the performance improvement. The average correlation coefficient results of the EMG signals post-processed by the 50 Hz digital notch filter were shown in Fig. 11. The EMG signals measured by the micropore insulated cEMG biomedical sensor and the wet contact electrode yielded the highest correlation coefficient value of 0.84 and 0.85 on subject A and subject B. Since the micropore insulated cEMG biomedical sensor originally has a relatively low noise floor, the 50 Hz digital notch filter does not bring a big improvement to the measurement results. The EMG signals measured by the gauze insulated cEMG biomedical sensor and the wet contact yielded the correlation coefficient value of 0.85 and 0.77 on subject A and subject B. The 50 Hz digital notch filter improved the correlation result of subject A by 0.01 and subject B by 0.06. The performance of a crepe bandage insulated cEMG biomedical sensor had the greatest improvement after the implementation of a 50 digital notch filter. The correlation coefficient of the EMG signals measured from subject A improved from 0.02 to 0.41 while subject B improved from 0.31 to 0.67. The 50 Hz digital notch filter was successfully attenuated the power line noise in the signal measurement.

Overall, the porous medical bandages were suitable to be used as an insulator of a cEMG biomedical sensor while different bandages will yield a different performance. A high input impedance cEMG biomedical sensor is highly susceptible to the environmental noise. A porous medical bandage insulated cEMG biomedical sensor which yielded a lower noise floor would eventually achieve a better EMG measurement result due to a better signal-to-noise ratio. A 50 Hz digital notch filter is proven to be able to effectively attenuate the dominant power line noise coupled into the cEMG biomedical sensor and improved the sensitivity of the sensor.

## Conclusion

A cEMG biomedical sensor has the flexibility to be insulated by different types of insulator. A porous medical bandage has the characteristics of hypoallergenic, breathable, and clinical approved material have made it a suitable candidate to be used as an insulator of a cEMG biomedical sensor. Different porous medical bandages will yield a unique skin–electrode capacitance and eventually affected the performance of a cEMG biomedical sensor. Among the evaluated porous medical bandages, micropore insulated cEMG biomedical sensor has the lowest noise floor of 2.44 mV and the highest correlation coefficient value of 0.83, in comparison with the conventional wet contact electrode. Since a high input impedance cEMG biomedical sensor is highly susceptible to the power line noise, a digital notch filter is proven can be effectively attenuate the power line noise and improve the performance of a porous medical bandages insulated cEMG biomedical sensor. These research outcomes will serve as an essential guideline for biomedical engineers and researchers to explore and advance the development of a medical grade porous cEMG biomedical sensor.
